# Horse and donkey owners’ perspectives on fireworks and their impact on equids in the UK

**DOI:** 10.1017/awf.2026.10068

**Published:** 2026-02-05

**Authors:** Stephanie L. Gerow, Simon R. Clegg, Andrew S. Cooke

**Affiliations:** School of Natural Sciences, Department of Life Sciences, University of Lincoln, Joseph Banks Laboratories, Beevor Street, Lincoln, LN6 7DL

**Keywords:** Animal welfare, behaviour, donkeys, equids, fear, fireworks, stress

## Abstract

In the UK, fireworks are common during several celebratory events throughout the year. Previous evidence has shown the adverse effects of fireworks on domestic companion animals. However, there has been little focus on equids. An online survey was developed to understand the impact of fireworks on horses and donkeys, how owners attempt to mitigate these impacts, and the owners’ views on fireworks. A total of 1,234 horse owners and 232 donkey owners responded. The majority (77%) advocated tighter regulations surrounding the use of fireworks, including reduction in the maximum noise produced, and control over when fireworks were used. Horse owners typically perceived their animals to be more fearful of fireworks than donkey owners, with running, kicking, bucking and rearing, being the most reported responses. However, horses used for hunting and sport were perceived as being less fearful. Eight percent of horse owners reported injury due to fireworks compared to donkeys, with only one report of injury. Stabling, staying with the animal, moving the animal to different premises, and music, were common mitigation strategies, all of which were rated as effective by owners. Owner concern and horse injury rates highlight fireworks as a potential threat to horse welfare and safety. Whilst owner mitigation strategies can be effective, they are limited in their ability to completely prevent injury and, importantly, require suitable forewarning. Differences between horses and donkeys are potentially due to different fear responses, with horses more likely to exhibit flight or fright responses, and donkeys flight or freeze.

## Introduction

In the UK, fireworks are predominantly used around November 5th (Guy Fawkes Night/Bonfire Night) and New Year’s Eve. To a lesser degree, they are also used at various other national, cultural, or religious events, such as the Twelfth of July (in Northern Ireland), Diwali, and Lunar/Chinese New Year, as well as for personal celebrations like weddings and birthdays.

The use of fireworks in England, Wales, and Northern Ireland is primarily regulated by the Fireworks Act 2003 and the Fireworks Regulations 2004 (Chapman *et al.* 2010). Scotland has similar legislation under the Fireworks and Pyrotechnic Articles (Scotland) Regulations 2004. The purpose of such legislation is to restrict the type of fireworks available to the public and to private displays, and to restrict the times of day and year during which fireworks can be used. Prevention of harm to animals (along with to people and property) is listed in these acts as a reason for their existence.

Fireworks are known to elicit fright responses in a range of domestic animals, including equids, particularly horses (Gronqvist *et al.* 2016; Gates *et al.* 2019; Dai *et al.* 2020; Lindstedt 2020). Gates *et al.* (2019) in New Zealand reported that 49.9% of horses expressed some form of fear behaviour in response to fireworks, with escaping being the most common response. Lindstedt (2020) studied horses’ behaviour during New Year’s Eve fireworks in Finland and reported stable location to be an important factor, with horses in urban environments being more likely to experience anxiety, which is complimented by Gronqvist *et al.* (2016) who suggests that stabling and moving of horses can be effective mitigation measures. Within the UK, horses are generally kept as animals for companionship, sport, or recreation, and less so for meat as may be the case elsewhere in the world. However, unlike smaller companion animals, such as cats and dogs, horses tend to live outside of the home and away from the owner. These circumstances can provide unique challenges for animal care as the owner cannot as readily observe how the animal responds to discrete but uncommon stimuli (i.e. fireworks) and thus not as easily react to the short-term needs of the animal in response to such stimuli. In the UK, whilst equids may be kept outdoors at night, this is reduced during the periods (i.e. winter) when fireworks are more common, meaning that they may be highly exposed to the audio and visual stimuli of fireworks, especially with the ability for sound to travel long distances over open ground (e.g. countryside).

Animal and horse charities and advocacy groups warn owners of the risk of fireworks to horses, e.g. “*As flight animals, horses can be particularly sensitive to the unpredictable loud noises and bright lights of fireworks displays”* (Redwings Horse Sanctuary 2025). They also offer advice, with Blue Cross (2025) suggesting that horses should be kept in a familiar environment, which could be outside, but that more frightened horses may need stabling. Between November 2010 and March 2024, The British Horse Society (2025) reported over a thousand incidents involving horses and fireworks, resulting in 35 deaths and 270 injuries – with reasonable likelihood that only a small proportion of incidents were reported. The organisation submitted evidence to the Office for Produce Safety and Standards, asking for a number of changes to be considered, including the use of silent fireworks in the vicinity of animals, a register for home firework use, a 500-m safe zone around horse dwellings, and advanced notice from display organisers to neighbours (The British Horse Society 2019). Calls for legislative and regulatory changes are strongest when supported by firm evidence. However, much of the evidence relating to the impact of fireworks on equids remains anecdotal and there is thus a need for targeted research to establish, and ideally quantify, the impacts.

### Study aim

The primary aim of this study was to understand the impact of fireworks on equids in the UK. This was achieved via an online survey distributed to UK horse and donkey owners. There were four sub-aims:

1. Assess horse and donkey owners’ opinions regarding the regulation of fireworks.
2. Quantify the behaviours that horses and donkeys exhibit in response to fireworks.
3. Report the type of injuries incurred by horses and donkeys as a direct or indirect result of fireworks.
4. Identify strategies that owners use to mitigate the risk and impact of fireworks on their animals and the self-reported efficacy of these strategies.

## Materials and methods

### Ethical considerations

This study received full ethical approval from the University of Lincoln Ethics Committee (reference number LEAS4279). All included respondents provided informed consent after reading the participant information sheet which outlined the purpose of the study and factors such as data storage and use. All owned either a horse or donkey, as signified by their answers to questions 11 and 12 (see Supplementary material). Respondents were all from the UK and were aged 18 or over. Participation was anonymous. Respondents could withdraw at any point whilst taking part, and once answers were submitted they could retrospectively withdraw as their results would not be identifiable.

### Survey

A survey (see Supplementary material) was distributed to UK horse and donkey owners. The survey was set up using the onlinesurveys.ac.uk (JISC) app, which allows for data collection with a high degree of data protection. People were invited to take part if they were owners of equids (either donkeys or horses) and lived within the UK. The survey was initially distributed via social media (Facebook and X [Twitter]) but was spread more widely using the snowball effect (Parker *et al.* 2019). The survey had five primary sections:

1. Owner background and views (Q1–10): This covered basic information regarding the owner (e.g. age, gender, area of living) and their general views on firework use and regulation.
2. About their equids (Q11, 22): Owners were asked a series of questions regarding their equid, including species, age, neuter status, use, and aspects of housing and management.
3. Behavioural responses to fireworks (Q23, 28): Owners reported the perceived fear level their equid had in response to fireworks. They also reported behaviours that they have observed their equid display in response to fireworks and the longevity of those behaviours.
4. Injuries (Q29, 33): Owners were asked if their animal had been injured by fireworks and, if so, details surrounding that injury, such as what the injury was, whether veterinary care was required, or if the animal died.
5. Mitigation (Q34, 38): Owners reported the mitigation strategies that they use to lessen the impact of fireworks on their animal and to score the effectiveness of these strategies from their experience. Owners were asked to report if they perceived the intervention to be very effective, moderately effective, somewhat effective, or ineffective most of the time.

### Statistical analysis

Due to the nature of the data gathered, such as much of it being ordinal, binary, categorical, or count data, non-parametric testing was opted for over parametric.

Though a direct comparison between horses and donkeys is not the primary purpose of the study, various comparisons were made as both sets of animal owners received the same questions, just with donkey/horse inserted where appropriate. Differences between owners’ perceptions of their horses and donkeys’ fear of fireworks were assessed using a Mann-Whitney *U* test. Differences in responses to fireworks were compared between horses and donkeys using a Chi-squared test with *P*-values adjusted for multiple comparisons using the Benjamini-Hochberg procedure. Differences in injury rates between horses and donkeys were made using a Chi-squared test.

Ordinal logistic regression models were fitted using the cumulative link model with a logit-link function to examine individual predictors of fear. Individual models were developed for horses and donkeys. The dependent variable was the owner-reported level of fear. For horses, the independent variables were sex and castration status (combined), age, origin, housing, location, environment, use/discipline, and the number of other horses at the location. For donkeys, the independent variables were size (full or miniature), sex and castration status (combined), age, origin, housing, location, use, and the number of other equids at the location. Regression models were followed by likelihood ratio testing to provide a summary of which factors (not levels within factors) were significantly associated with fear.

Variations in owner-perceived fear in their animals were compared across different display types that the owners had reported in their area. This was completed independently for both horses and donkeys using a Kruskal-Wallis test; where that test yielded a statistically significant result, a *post hoc* Dunn’s test was performed, with the Benjamini-Hochberg procedure applied to adjust for multiple comparisons.

## Results

### Sample summary

A total of 1,466 owners responded to the survey, this comprised of n = 1,234 horse owners and n = 232 donkey owners. Respondents were predominantly female, and there was a distribution of ages. Characteristics of equids varied and they represented a variety of sex, age, origin, castration status, housing, and environments (Table 1).

**Table 1.** Summary of the equid population sampled and their environment, split between horses (n = 1,234) and donkeys (n = 232). “Origin” refers to where the owner obtained the animal. “Housing” refers to the type of yard/place where the animal lives. “Environment” refers to the type of environment where the yard is located

		Horses		Donkeys	
		%	n/n	%	n/n
Sex	Female	57.6%	711/1,234	54.7%	127/232
	Male	42.4%	523/1,234	45.3%	105/232
Age (years)	≤5	17.8%	220/1,234	9.9%	23/232
	6–10	29.5%	364/1,234	27.2%	63/232
	11–20	31.2%	385/1,234	34.5%	80/232
	>20	21.5%	265/1,234	28.4%	66/232
Origin	Breeder	15.6%	193/1,234	10.8%	25/232
	Family	20.9%	258/1,234	19.4%	45/232
	Rehomed	24.1%	298/1,234	22.8%	53/232
	Rescue	26.7%	330/1,234	35.8%	83/232
	Other	12.6%	155/1,234	11.2%	26/232
Castrated^*^	Yes	63.5%	337/523	57.1%	60/105
	No	36.5%	186/523	42.9%	45/105
Housing	Farm	32.5%	284/1,234	34.5%	80/232
	Livery yard	23.0%	401/1,234	13.8%	32/232
	Private dwelling	31.1%	384/1,234	34.9%	81/232
	Other	13.4%	165/1,234	16.8%	39/232
Environment	Urban	14.4%	176/1,234	20.3%	47/232
	Suburban	38.8%	479/1,234	32.3%	75/232
	Rural	46.9%	579/1,234	47.4%	110/232

* only applies to males.

### Views towards fireworks

A greater proportion of horse owners (34.4%; 425/1,234) believed that fireworks should be banned, compared to donkey owners (15.1%; 35/232). However, the proportion of owners who felt that tighter regulations were needed were similar in both groups, as were their views regarding how regulations should change, with a restriction of firework use to specific occasions being the most common answers (Table 2).

**Table 2.** UK equid owners’ views towards fireworks and their regulation

Question	Answer	Horse owners		Donkey owners	
		%	n/n	%	n/n
Fireworks should be banned	Yes	34.40%	425/1,234	15.10%	35/232
	No	51.30%	633/1,234	70.30%	163/232
	Don’t know	14.30%	176/1,234	14.70%	34/232
Tighter regulations are needed	Yes	76.70%	947/1,234	80.60%	187/232
	No	23.30%	287/1,234	19.40%	45/232
What should be changed^*^	Only organised displays	29.90%	285/1,014	29.60%	47/160
	Only for certain occasions	37.90%	344/1,234	39.00%	62/160
	Reduce maximum noise	32.20%	301/1,234	31.40%	50/160

* Results only represent those who answered “Yes” when asked if there should be tighter regulations.

### Behaviour and responses

**Figure 1.**
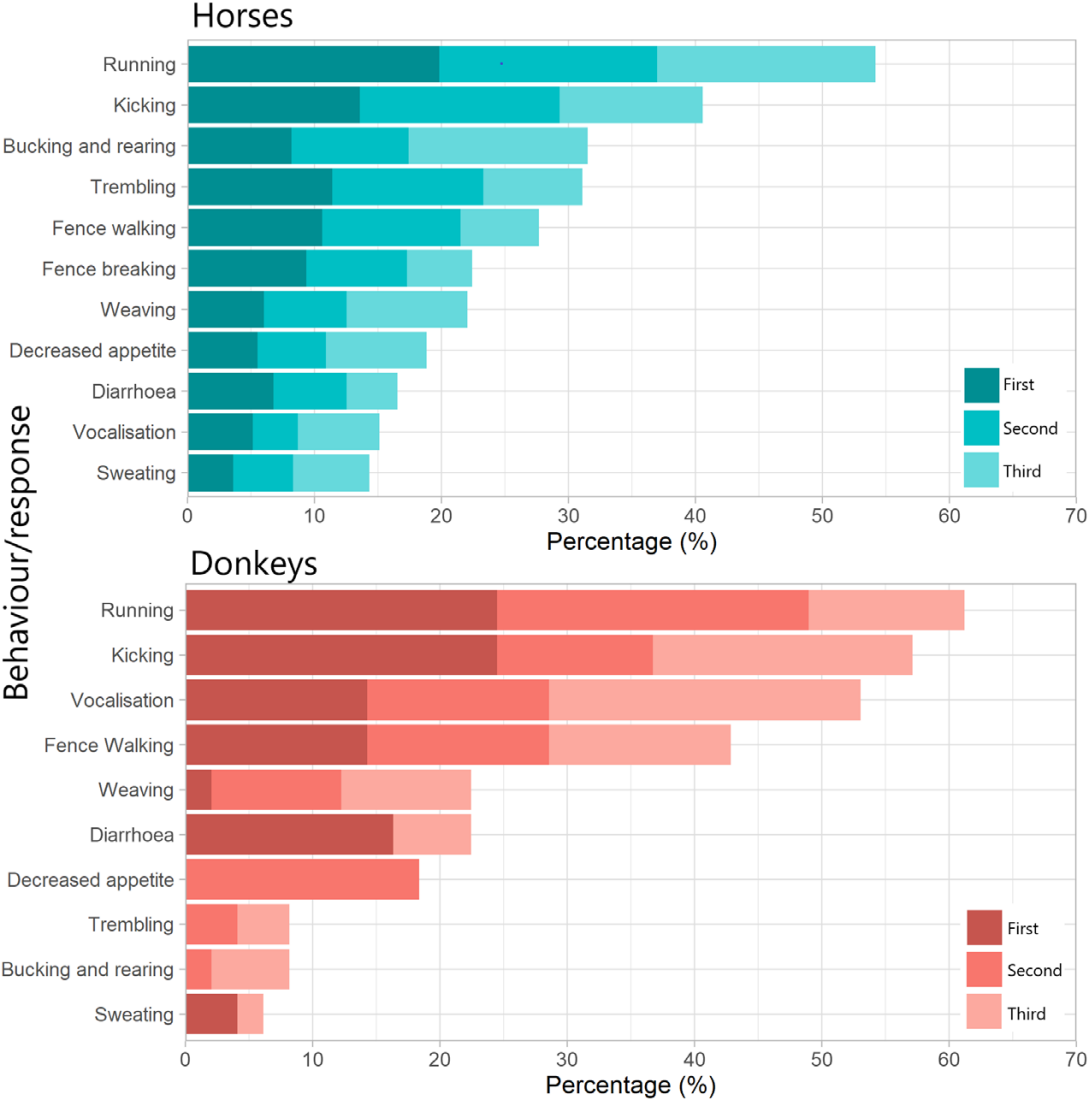
Percentage of owners that reported specific behaviours and responses from their horse or donkey in response to fireworks. Results consist only of those from owners answering ‘Yes’ to the question ‘Does your [horse/donkey] respond negatively to fireworks?’ (Horses; n = 781, Donkeys; n = 49). ‘First’, ‘Second’ and ‘Third’ refer to whether that specific behaviour is the first, second, or third most common behaviour reported by that owner regarding their animal (bars are stacked). N.B. these results represent all owners, and certain activities may only be possible under specific circumstances, e.g. a stabled horse cannot display ‘fence-walking’.

The majority (63.3%; 781/1,234) of horse owners stated that their horses responded negatively to fireworks, compared to a minority (21.1%; 49/232) of donkey owners. There was a significant difference in the prevalence of responses between horses and donkeys (Table 3). For both animals, running and kicking were the most commonly reported behaviours (Figure 1). Bucking and rearing was relatively common in horses, but uncommon in donkeys. Conversely, vocalisation was not commonly reported for horses yet was reported for the majority of donkeys. Though diarrhoea was only somewhat common in both groups, for a subset of donkeys it appeared to be the predominant response.

**Table 3.** Chi-squared test results comparing the frequency of behaviours and responses to fireworks between horses and donkeys. P-values have been adjusted for multiple (10) comparisons using the Benjamini-Hochberg procedure

Behaviour/response	χ^2^	*P-*value
Running	3.069	0.423
Kicking	9.655	0.044
Bucking and rearing	12.456	0.016
Trembling	12.353	0.016
Fence walking	7.152	0.096
Weaving	2.21	0.53
Decreased appetite	19.25	0.001
Diarrhoea	9.25	0.044
Vocalisation	47.587	<0.001
Sweating	3.975	0.33

### Fear

**Figure 2.**
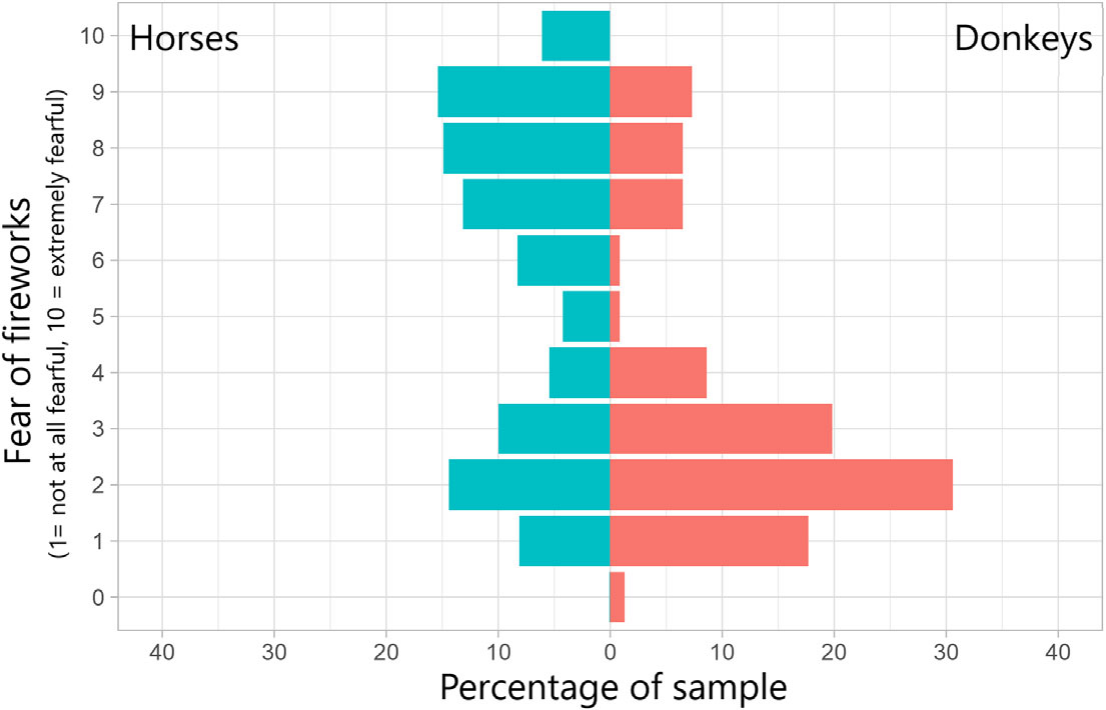
Split histogram showing owners’ perceptions regarding their animals’ fear levels as a result of fireworks. Percentages are independent for horses and donkeys. The x-axis represents the owner’s perception of their animals fear level regarding fireworks, scored from 1 (not at all fearful) to 10 (extremely fearful). Bars represent the percentage of owners, for each equid group, that reported a specific score.

Horse owners typically rated their animals as being more scared of fireworks than donkey owners, with a median scores of 6 and 3 recorded for horses and donkeys, respectively (W = 83,893; *P* < 0.001) (Figure 2).

For horses, likelihood ratio testing found ‘use’ to be the only factor that was significantly associated with fear (Tables 4–6). The full output for ordinal regression models can be found in Tables S1 and S2 in the Supplementary material. Fear score levels were significantly lower for horses used for hunting (x̄ = 2.0, *z* = -9.489; *P* < 0.001) and sport (x̄ = 3.7, *z* = –3.808; *P* < 0.001), and significantly higher for horses used for pony club (x̄ = 6.7, *z* = 4.066; *P* < 0.001) and pleasure (x̄ = 6.7, *z* = 4.028; *P* < 0.001). For donkeys, likelihood ratio testing found other equids to be significantly associated with fear of fireworks, with ordinal regression revealing that donkeys housed in the presence of a greater number of equids had lower owner-reported levels of fear of fireworks (z = –2.428; *P* = 0.152). Though not shown in the likelihood ratio testing, ordinal regression did find that donkeys originating from ‘other’ (e.g. not rehomed, rescue, from family, or breeders) scored higher in terms of fear level (x̄ = 4.2, z = 2.302; *P* = 0.021).

**Table 4.** Statistical output (χ^2^ and *P*-value) from likelihood ratio testing (after ordinal regression), showing the association of individual animal factors with owner-perceived fear of fireworks in their animals. Analysis for horses and donkeys were performed independently

Species	Factor	χ^2^	*P-*value
Horses	Sex and neutering	4.061	0.255
	Age	4.234	0.237
	Origin	3.797	0.434
	Yard	2.613	0.455
	Location	1.192	0.551
	Use	237.241	<0.001
	Others	1.082	0.298
Donkeys	Size	0.701	0.704
	Sex	2.347	0.309
	Age	3.388	0.336
	Origin	6.192	0.185
	Housing	1.525	0.676
	Location	0.048	0.977
	Use	3.482	0.323
	n other equids	5.935	0.015

**Table 5.** Results for ordinal regression assessing predictors of fear of fireworks in horses.

Horses						
Variable	Level	Estimate	z-value	*P*-value	Odds ratio	95% CI
Sex	Female (entire)	Reference category				
	Male (entire)	–0.061	–0.414	0.679	0.941	0.705–1.256
	Female (neutered	1.651	1.958	0.05	5.213	0.999–27.213
	Male (castrated	–0.016	–0.133	0.894	0.984	0.782–1.240
Age	≤5 years	Reference category				
	6–10 years	0.234	1.532	0.126	1.264	0.937–1.706
	11–20 years	–0.011	–0.075	0.94	0.989	0.733–1.333
	>20 years	0.042	0.228	0.82	1.042	0.729–1.491
Origin	Breeder	Reference category				
	Family	–0.257	–1.472	0.141	0.773	0.549–1.089
	Other	0.036	0.185	0.853	1.037	0.708–1.519
	Rehomed	–0.043	–0.257	0.797	0.957	0.687–1.333
	Rescue	–0.06	–0.36	0.719	0.942	0.681–1.303
Yard	Farm	Reference category				
	Livery yard	–0.254	–1.297	0.195	0.776	0.529–1.138
	Other	0.073	0.412	0.68	1.076	0.759–1.525
	Private dwelling	–0.07	–0.477	0.634	0.932	0.698–1.244
Location	Rural	Reference category				
	Suburban	0.101	0.898	0.369	1.106	0.887–1.379
	Urban	–0.044	–0.277	0.782	0.957	0.701–1.306
Use	Breeding	Reference category				
	Competitions	0.036	0.177	0.86	1.037	0.693–1.551
	Endurance	0.145	0.571	0.568	1.156	0.702–1.903
	Hunting	–3.009	–9.489	<0.001	0.049	0.027–0.092
	Pleasure	0.823	4.028	<0.001	2.277	1.526–3.399
	Pony club	0.936	4.066	<0.001	2.549	1.623–4.001
	Racing	–0.641	–1.683	0.092	0.527	0.250–1.111
	Sports	–0.907	–3.808	<0.001	0.379	0.230–0.624
Others	n other	0.004	1.04	0.298	1.004	0.996–1.013

*Note:* one level of each variable is a reference category

**Table 6.** Results for ordinal regression assessing predictors of fear of fireworks in donkeys.

Donkeys						
Variable	Level	Estimate	z-value	*P*-value	Odds ratio	95% CI
Sex	Female (entire)	Reference category				
	Male (entire)	–0.354	–1.05	0.294	0.702	0.362–1.360
	Male (castrated)	–0.4	–1.323	0.186	0.671	0.371–1.212
Age	≤5 years	Reference category				
	6 – 10 years	–0.259	–0.556	0.578	0.772	0.310–1.923
	11– 20 years	0.151	0.328	0.743	1.163	0.472–2.866
	>20 years	–0.365	–0.762	0.446	0.694	0.271–1.776
Origin	Breeder	Reference category				
	Family	0.814	1.797	0.072	2.258	0.929–5.490
	Other	1.124	2.302	0.021	3.079	1.182–8.020
	Rehomed	0.461	1.039	0.299	1.586	0.664–3.788
	Rescue	0.59	1.432	0.152	1.805	0.804–4.049
Yard	Farm	Reference category				
	Livery yard	–0.323	–0.73	0.465	0.724	0.304–1.723
	Other	–0.423	–0.97	0.332	0.655	0.279–1.539
	Private dwelling	–0.059	–0.153	0.878	0.943	0.443–2.004
Location	Rural	Reference category				
	Suburban	–0.016	–0.046	0.963	0.984	0.491–1.972
	Urban	0.06	0.15	0.881	1.062	0.486–2.317
Use						
	Other	–2.157	–1.652	0.098	0.116	0.009–1.494
	Pet/Companion	–0.681	–0.921	0.357	0.506	0.119–2.157
	Working	–0.929	–1.232	0.218	0.395	0.090–1.731
Size	Both	Reference category				
	Full	0.226	0.778	0.436	1.253	0.710–2.213
	Mini	0.262	0.67	0.503	1.299	0.604–2.793
Others	n others	–0.142	–2.428	0.015	0.867	0.773–0.973

*Note:* one level of each variable is a reference category

**Figure 3.**
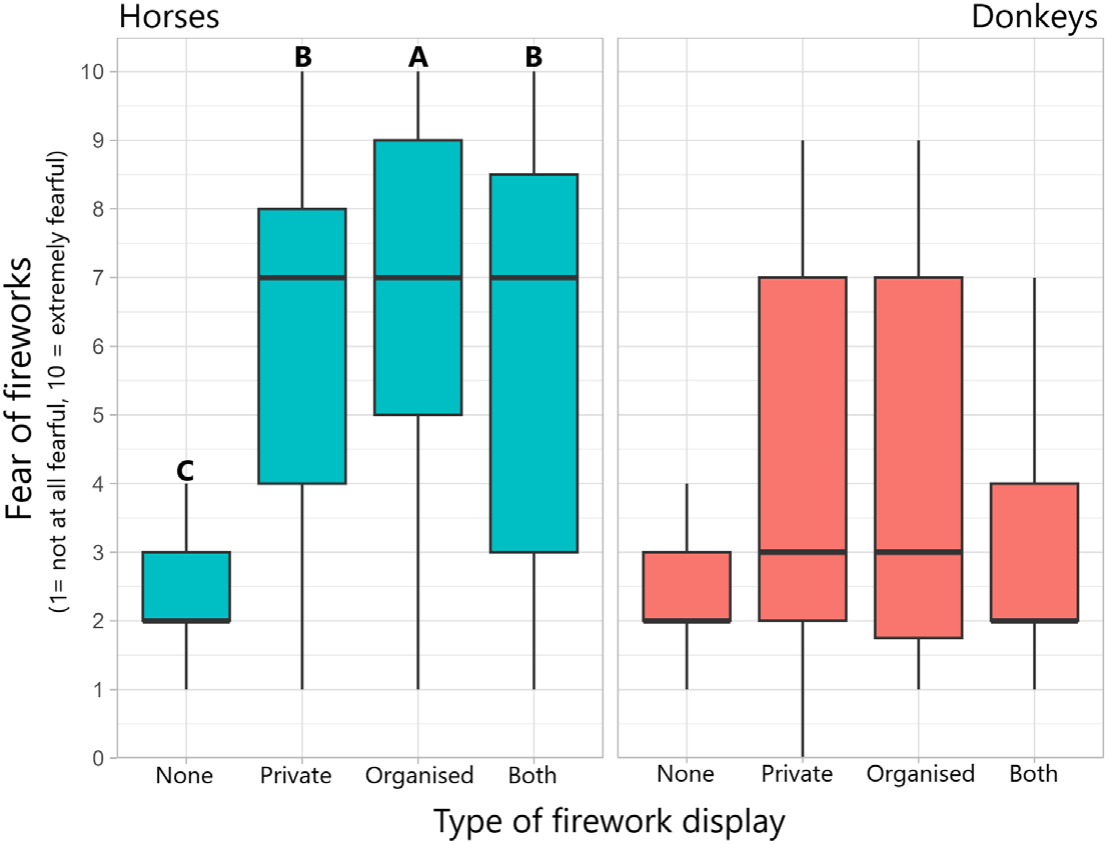
Owner-reported equid fear levels of fireworks shown by species and compared across different types of fireworks displays reported near the animals’ housing. Boxplots represent the median (central line), first and third quartiles (bottom and top of boxes) and range (extent to which the lines extend). Superscript letters are derived from *post hoc* Dunn’s tests after a Kruskal-Wallis test. A Dunn’s test was only performed for horses as the Kruskal-Wallis test for donkeys did not show a statistically significant difference. For horses, boxes featuring the same superscript do not differ significantly while those with different superscripts differ significantly from one another. Paired results for Dunn’s tests were: None vs Private (*z* = –12.9; *P* < 0.001); None vs Organised (*z* = –14.1; *P* < 0.001); None vs Both (*z* = –11.6; *P* < 0.001); Private vs Organised (*z* = 2.2; *P* = 0.019); Private vs Both (*z* = 0.9; *P =* 0.190); and Organised vs Both (*z* = 2.8; *P* = 0.003).

The type of fireworks displays that owners reported in their area had an impact upon the perceived level of fear in horses (χ^2^ = 225.47; *P* < 0.001) but not donkeys (χ^2^ = 2.89; *P* = 0.409) (Figure 3). For horses, greater fear levels were reported for animals housed near organised displays, compared to those housed close to private displays, or both.

When asked to provide the aspects of fireworks which proved most problematic for their animals, horse owners most commonly cited ‘loud bangs’ (35.3%; 435/1,234) followed by ‘flashing lights’ (21.6%; 267/1,234), however a moderate number reported ‘none’ (28.1%; 347/1,234). For donkey owners, the majority stated ‘none’ (78.4%; 182/232), but some did also cite loud bangs (10.8%; 25/232) and flashing lights (6.5%; 15/232). ‘Falling debris’ and ‘falling embers’ were only reported by a small number of horse owners (6.3% [78/1,234] and 2.9% [36/1,234], respectively), whilst ‘falling embers’ was only reported by one donkey owner and falling debris was reported by none.

### Injury

Injuries associated with fireworks were reported by 8.1% (100/1,234) of horse owners. Of these, the majority were cuts/lacerations (79%; 79/100). Other injuries included broken bones (11%; 11/100), hoof and foot damage (5%; 5/100), car accidents after escaping (3%; 3/100), abrasions (1%; 1/100), damage to teeth (1%; 1/100), and eye damage from a tree (1%; 1/100). Veterinary care was required for 51% (51/100) of injuries. Of the reported injuries to horses, 12% (12/100) resulted in death, nine from broken bones, and three from car accidents. Only one donkey owner reported their animal being injured by fireworks, stating that the animal experienced ‘cuts’, which did not require veterinary care. Injury rates differed significantly between horses and donkeys (*χ* ^2^ = 17.071; *P* < 0.001). Of horse owners reporting injuries, 45% (45/100) reported that the horse had managed to escape from its housing, though not asked to clarify if this was because of fireworks, this is assumed to be the case given the implication and context of the question.

### Mitigation

Some form of mitigation strategy was used by 74.8% (923/1,234) of horse owners and 20.7% (48/232) of donkey owners. For both groups, the most common strategy entailed ensuring that the animal is stabled. The strategy deemed to be most effective was to move the animal from the premises. Horse owners appeared to employ a broader range of mitigation strategies, although the sample size is larger than for donkeys (Table 7).

**Table 7.** Owner-reported strategies for mitigating the risk of fireworks to their horses or donkeys, chosen from a set list of strategies. Percentages are proportional to the number of owners who employed some form of mitigation (74.8% [923/1234] of horse owners, 20.7% [48/232] of donkey owners)

Mitigation	Horses	Donkeys	Median effectiveness (1–4)	% responses	Median effectiveness (1–4)	Median effectiveness (1–4)
	%	n/n				
Stabled	37.10%	362/923	3	70.80%	12/48	3
Stay with animal	24.50%	226/923	3	18.80%	9/48	3
Move off premises	11.20%	103/923	4	2.10%	1/48	4
Complete routine early	8.10%	75/923	4	2.10%	14/48	2
Sedation	6.90%	64/923	3	0.00%	0/48	-
Music	4.90%	45/933	3	6.30%	3/48	3
Leave lights on	4.40%	41/923	3	0.00%	0/48	-
Noise-reducing headwear	1.80%	17/923	2	0.00%	0/48	-
Additional food	0.70%	6/923	3.5	0.00%	0/48	-
Other	0.40%	4/923	3.5	0.00%	0.48	-

### Discussion

For both species, the distribution of scores for fear of fireworks was bimodal. This either suggests that fear responses to fireworks are generally very muted or very strong, with little in between or, that the owners’ reports of fear of fireworks were somewhat binary (e.g. scared or not), creating a split in the sample. This is supported by Lansade *et al.* (2008), who investigated a ‘fearfulness trait’ in horses; and concluded that those classified as more fearful responded more strongly to novel sounds, suggesting certain horses are inherently more vulnerable to distress. In addition, Riva *et al.* (2022) reported that horses generally fell into one of two categories – very anxious or slightly anxious, which fits with this bimodal pattern. The greater injury rate observed in horses may be a consequence of their increased fear towards fireworks, which may have triggered erratic and panicked behaviour, evidenced here by increased bucking and rearing and fence-breaking. However, this evidence is circumstantial without direct observational evidence. The combination of these factors may also explain why horse owners were more inclined than donkey owners to implement mitigating strategies.

Horses that were used for hunting and sport had lower scores for fear. This may be due to a greater familiarity with unexpected and loud sounds, which may have enabled them to develop a resilience to novel stimuli and stressors (Hole *et al.* 2023). This suggests a capacity for horses to become acclimated to loud noises through lifetime exposure, though further evidence is required to establish this effect. Conversely, horses used for Pony Club and Pleasure showed higher scores regarding fear of fireworks. An alternative explanation may be that the owners of these horses differ in their perception of horses’ fear and other affective states. Another notable finding was that scores for fear were greater for horses close to organised fireworks displays, as opposed to private ones. This is potentially due to the greater size and quantity of fireworks within organised displays, compared to private displays.

Donkeys had lower scores for fear when accompanied by a greater number of other equids. Donkeys can form particularly strong social bonds and, whilst these bonds are not fully understood, they may contribute towards stress mitigation (Murray *et al.* 2013; Burden & Thiemann 2015). The higher fear scores for donkeys originating from ‘other’ sources (which represented 11.2% of responses) are unclear, as owners were not asked to clarify as to what ‘other’ referred.

Results displayed similarities with those of Gronqvist *et al.* (2016), who reported relatively high incidences of running, fence-breaking, and injuries. However, they also reported stabling and moving horses as the most common strategies of mitigation at 55 and 77%, respectively, levels far higher than reported here. Gates *et al.* (2019) reported a high rate of escape (73%) for horses and that owners’ most common mitigation strategies included comforting the animal and moving the animal inside/outside (interestingly, an even split in that choice). Results from Gates *et al.* (2019) and Gronqvist *et al.* (2016) are in broad accordance with ours, although both studies took place in New Zealand which shares cultural similarities with the UK, but reduced population densities. The latter point potentially influential when taking into consideration both the frequency of firework use and the equids’ familiarity with anthropogenic activity.

The differences between horses and donkeys may be a direct result of differences in their nature and temperament. When frightened or in pain, donkeys typically exhibit more subtle behaviours than horses (Burden & Thiemann 2015), often being referred to as ‘stoic’, although this does not necessarily equate to them experiencing less fear or pain. Vidament *et al.* (2023) reported that donkeys slowed down or refused to move when facing novelties. This could suggest that a key defence mechanism of donkeys is to remain static (freeze) to avoid being noticed, whereas horses may be more inclined to try and escape (flight). Prescott *et al.* (2022) reported donkeys as being more likely to have ‘fight’ or ‘freeze’ responses to fear and threats, compared to horses that were more likely to show a ‘flight’ response. Donkeys have also been reported to respond more actively to visual cues than auditory (Gonzalez-De Cara *et al.* 2017), although no studies have directly compared this with horses as yet. However, Christensen *et al.* (2005) reported that horses showed greater responses (steps backwards) to novel auditory stimuli than novel visual stimuli.

Mitigation strategies reported by owners varied. To some extent, this reflected the causes of injury that were reported. For example, stabling was the most commonly described mitigation strategy, which could reduce the chance of escape (and thus car accidents), injury on trees and fences, and limit the potential for the animal to gather enough speed for a serious accident on potentially uneven ground. However, the benefits and risk of this are yet to be studied and confining a frightened animal carries the risk of injury. The strategy of moving equids to different premises was deemed most effective, which is complemented by results from Lindstedt (2020), who reported an association between stable location and firework noise anxiety. This is similar to the results shown by Gronqvist *et al.* (2016) in New Zealand, who reported moving horses away from an area affected by firework noise, or stabling them as the commonest strategies deployed and those considered the most effective in terms of reducing the negative effects of fireworks on equine behaviour. However, such strategies must be balanced against the risks of harm and stress that could occur during their implementation. Moving horses or providing hay were the two management strategies suggested to be effective against anxiety behaviours reported in another UK-based study (Riva *et al.* 2022), although this study broke the horses into a very anxious and slightly anxious group, which our study did not.

Some owners (24.5% of horse owners, and 18.8% of donkey owners) reported that their main mitigation strategy entailed staying with their equid, and while this may prove effective, it does expose the owner to the risk of physical injury from spontaneous reactions by the horse, such as bucking. Further studies may be required assessing other interventions, with some studies showing that music can have a relaxing effect on horses (Fiedler & McGreevy 2016) and that noise dampening ear defenders may also help to reduce noise-induced, anxiety-based behaviours (Hole *et al.* 2023). Other studies have reported the positive effects various drugs can have on reducing firework anxiety in horses, with detomidine oromucosal gel being highlighted as one to show potential success in mitigating anxiety behaviour (Dai *et al.* 2020).

When owners were asked what legislative changes they would wish to see, the most common response was for fireworks to be restricted for use on special occasions only. Having specific dates during which fireworks could only be used would allow owners to prepare and minimise the risk to their animals. Stronger views were more prevalent from horse owners compared to donkey owners, possibly reflecting the higher injury rate and perceived fear that was reported. This is in line with recent UK Parliamentary debates regarding firework regulations, in particular those which make large amounts of noise, which are likely to be the ones which cause issues for the equids in this study (change.org 2018; Uwazuruike *et al.* 2025).

### Study limitations

There were several limitations, most notably, fear scores were supplied by the owners themselves and not measured directly. Thus, differences in owner perceptions may have impacted scores provided, in particular the contrast seen between different horse uses/disciplines. Whilst owners were asked for their views regarding the tightening of firework regulations, they did not have opportunity to express an opinion in favour of relaxing firework regulations.

Recognition of fear, and other affective states, in equids is limited and people commonly fail to recognise signs of distress (Bell *et al.* 2019), especially the more subtle signs (Rogers & Bell 2022). Equine stakeholders have also identified recognition and interpretation of stress and pain-associated behaviour to be a potential welfare issue (Horseman *et al.* 2016). Lower fear scores for donkeys, compared to horses, may not necessarily be because they are less fearful, but may be because the manifestation of that fear, especially freeze responses, may not be interpreted by owners as fear. Another limitation is that recruitment was performed online via a variety of animal and equine charities and advocacy groups. Members of the public that interact with these groups online may be more engaged in equine welfare than the wider equid-owning population. Finally, many owners do not live with their animal and therefore may not be aware of the response of their horse to fireworks, especially if it is a temporary behavioural response as opposed to any form of physical harm.

This study does show that equids within the UK are subjected to additional stress and stress-induced behaviours largely due to fireworks and the noises and the visual effects which they generate. These issues appear to be more of an problem for horses than for donkeys and raises the question of whether co-habiting a donkey with a horse would have an altered effect. Clearly, many owners were proactive and took mitigating steps to protect their horses, however spontaneous, unnotified firework events pose a risk to animal welfare. These results are most generalisable to the UK, however, will still be somewhat applicable to other locations where horse management and the annual calendar of firework usage is broadly similar.

### Animal welfare implications

Findings highlight animal welfare concerns associated with the use of fireworks in proximity to equids, particularly horses. Owners reported a variety of fear responses in their equids, which were manifested by behaviours, such as bucking and rearing, that could be indicative of distress. Owners also reported injuries and even deaths of horses in relation to fireworks. Whilst many owners employed strategies to mitigate these risks, such measures are not always feasible or effective, especially when fireworks are unexpected. This evidence can contribute to supporting and educating owners regarding the risks their animals may face and how best to manage them. The observed differences between horses and donkeys suggest that guidance and management strategies should be specifically tailored to each species. These findings may also inform future legislation by providing evidence of the potential harm caused by fireworks to equine welfare.

## Conclusion

Based on owner reports, horses appear to be at a greater risk of injury and harm from fireworks than donkeys. These injuries are typically indirect, caused by the horse’s reaction to fireworks, not by the fireworks themselves. Donkeys’ responses to fireworks appear to be less extreme, reducing the risk of injury. However, it was not discerned if this is because they are less fearful of fireworks or because they employ a ‘freeze’ response to them. A range of mitigation strategies were self-assessed by owners as effective in limiting the impact of fireworks on equids and include ensuring that the animal is kept in a well-illuminated, secure location, with no possibility to escape, a lack of objects which can cause harm, and if possible, away from firework displays. However, mitigation strategies must be balanced against the stress of their implementation, which will be unique to the circumstances of each owner and animal.

## Supporting information

10.1017/awf.2026.10068.sm001 Gerow et al. supplementary material Gerow et al. supplementary material

## Supplementary material

The supplementary material for this article can be found at http://doi.org/10.1017/awf.2026.10068.
