# Supplementary material for: Horse and donkey owners’ perspectives on fireworks and their impact on equids in the UK
Source: Anim Welf. 2026 Feb 5;35:e11. doi: 10.1017/awf.2026.10068 (PMC12895199; doi:10.1017/awf.2026.10068)
Supplement: Gerow et al. supplementary material [file S0962728626100682sup001.pdf]

Horse and donkey owners' perspectives on fireworks and their impact on equids in the UK: Supplementary material

Stephanie L Gerow, Simon R Clegg, Andrew S Cookehttps://orcid.org/0000-0003-2225-1890

School of Natural Sciences, Department of Life Sciences, University of Lincoln, Joseph Banks Laboratories, Beevor Street, Lincoln LN6 7DL, UK

Author for correspondence: Simon R Clegg, email: sclegg@lincoln.ac.uk

Survey questions

1. What is your age?
   a. Under 20
   b. 21-40
   c. 41-60
   d. 61-80
   e. Over 80
2. What is your gender?
   a. Female
   b. Male
   c. Other
3. In what type of area do you live?
   a. Rural
   b. Suburban
   c. Urban
4. Do any children under the age of 16 live in the household?
   a. Yes
   b. No
5. Do you buy fireworks (other than sparklers)?
   a. Yes
   b. No
6. Do you attend public firework displays?
   a. Yes
   b. No
7. Do you think fireworks should be banned?
   a. Yes
   b. No
   c. Don't know
8. Are tighter regulations needed on fireworks?
   a. Yes
   b. No

9. How should fireworks regulations be tightened?
    a. No change needed
    b. Only for use on certain occasions
    c. Only for use in organised displays
    d. Reduced maximum noise
    e. Total ban
10. When is the most common time that you hear fireworks?
    a. Around November 5th
    b. Around Christmas and New Year
    c. November and December
    d. Regularly
    e. Never
11. Do you own a horse?
    a. Yes
    b. No
12. Do you own a [horse / donkey]?
    a. Yes
    b. No
13. Is it full-sized or miniature? [donkey owners only]
    a. Mini
    b. Full
    c. Both
14. What is the sex of your [horse / donkey]?
    a. Female
    b. Male
15. What is the approximate age of your [horse / donkey]?
    a. 5 years or less
    b. 6-10 years
    c. 11-20 years
    d. More than 20 years
16. Is the [horse / donkey] neutered?
    a. Yes
    b. No
17. Where did you obtain the [horse / donkey] from?
    a. Breeder
    b. Family
    c. Rehomed
    d. Rescue
    e. Other
18. Where is the [horse / donkey] usually located?
    a. Farm
    b. Livery yard
    c. Private dwelling
    d. Other
19. In what sort of area is the [horse / donkey] located?
    a. Rural
    b. Suburban
    c. Urban
20. How close is your home is the [horse / donkey] located?
    a. Less than 2 miles
    b. 2-5 miles
    c. Over 5 miles
21. Approximately how many [horses / donkeys] are on the same site as your [horse / donkey]?
    a. *Typed numerical answer*
22. What is the main use of your [horse / donkey]? *Answers supersriptted with "h" were only available to horse owners and "d" only available to donkey owners.*
    a. Breeding
    b. hCompetition

c. [h]Endurance
d. [h]Hunting
e. [d]Pet/companion
f. [h]Pleasure
g. [h]Pony club
h. [h]Racing
i. [h]Sports
j. [d]Working

23. One a scale of 1-10 how frightened is your [horse / donkey] of fireworks?
    a. 1
    b. 2
    c. 3
    d. 4
    e. 5
    f. 6
    g. 7
    h. 8
    i. 9
    j. 10
24. Does your [horse / donkey] respond negatively to fireworks?
    a. Yes
    b. No
25. What is the most common behaviour your [horse / donkey] shows from fireworks (if any)?
    a. Breaking fences
    b. Bucking and rearing
    c. Decreased appetite
    d. Diarrhoea
    e. Fence walking
    f. Kicking
    g. Running
    h. Sweating
    i. Trembling
    j. Vocalisation
    k. Weaving
26. What is the second most common behaviour your [horse / donkey] shows from fireworks (if any)?
    a. Breaking fences
    b. Bucking and rearing
    c. Decreased appetite
    d. Diarrhoea
    e. Fence walking
    f. Kicking
    g. Running
    h. Sweating
    i. Trembling
    j. Vocalisation
    k. Weaving
27. What is the third most common behaviour your [horse / donkey] shows from fireworks (if any)?
    a. Breaking fences
    b. Bucking and rearing
    c. Decreased appetite
    d. Diarrhoea
    e. Fence walking
    f. Kicking
    g. Running
    h. Sweating
    i. Trembling
    j. Vocalisation
    k. Weaving

28. How long do these behaviours normally last?
    a. Into the day after
    b. Just during the noise
    c. Longer than 24hrs
    d. The rest of the night
    e. Up to 2hrs
29. Has your [horse / donkey] injured itself due to fireworks?
    a. Yes
    b. No
30. What injuries were seen?
    a. *Free text*
31. Did the injury require veterinary intervention?
    a. Yes
    b. No
32. Did the [horse / donkey] die due to this injury?
    a. Yes
    b. No
33. Did the [horse / donkey] escape from its housing?
    a. Yes
    b. No
34. What management strategies do/did you use for your [horse / donkey]?
    a. Complete routing earlier
    b. Leave the lights on
    c. Move animal off the property
    d. Music
    e. Noise reducing headwear
    f. Nothing
    g. Other
    h. Provide additional food
    i. Sedation
    j. Stabling
    k. Staying with the horse
35. How effective are these measures in preventing the reported behaviours?
    a. Ineffective
    b. Somewhat effective
    c. Moderately effective
    d. Very effective
36. How often do you hear fireworks where the [horse / donkey] is kept?
    a. Around November 5th
    b. Around Christmas and New Year
    c. November and December
    d. Regularly
    e. Never
37. Which aspects of fireworks causes problems for your [horse / donkey]?
    a. Crackles
    b. Falling debris
    c. Falling embers
    d. Flashing lights
    e. Loud bangs
    f. None
38. What type of fireworks displays are heard where your [horse / donkey] is housed?
    a. None
    b. Private displays
    c. Pubic displays
    d. Both

Original regression

Table S1 - Results for ordinal regression assessing predictors of fear of fireworks in horses. Note: one level of each variable is a reference category.

**Horses**						
Variable	Level	Estimate	z-value	p-value	Odds ratio	95% CI
Sex	Female (entire)	Reference category				
	Male (entire)	-0.061	-0.414	0.679	0.941	0.705 - 1.256
	Female (neutered	1.651	1.958	0.050	5.213	0.999 - 27.213
	Male (castrated	-0.016	-0.133	0.894	0.984	0.782 - 1.240
Age	≤5 years	Reference category				
	6 - 10 years	0.234	1.532	0.126	1.264	0.937 - 1.706
	11- 20 years	-0.011	-0.075	0.940	0.989	0.733 - 1.333
	>20 years	0.042	0.228	0.820	1.042	0.729 - 1.491
Origin	Breeder	Reference category				
	Family	-0.257	-1.472	0.141	0.773	0.549 - 1.089
	Other	0.036	0.185	0.853	1.037	0.708 - 1.519
	Rehomed	-0.043	-0.257	0.797	0.957	0.687 - 1.333
	Rescue	-0.060	-0.360	0.719	0.942	0.681 - 1.303
Yard	Farm	Reference category				
	Livery yard	-0.254	-1.297	0.195	0.776	0.529 - 1.138
	Other	0.073	0.412	0.680	1.076	0.759 - 1.525
	Private dwelling	-0.070	-0.477	0.634	0.932	0.698 - 1.244
Location	Rural	Reference category				
	Suburban	0.101	0.898	0.369	1.106	0.887 - 1.379
	Urban	-0.044	-0.277	0.782	0.957	0.701 - 1.306
Use	Breeding	Reference category				
	Competitions	0.036	0.177	0.860	1.037	0.693 - 1.551
	Endurance	0.145	0.571	0.568	1.156	0.702 - 1.903
	Hunting	-3.009	-9.489	<0.001	0.049	0.027 - 0.092
	Pleasure	0.823	4.028	<0.001	2.277	1.526 - 3.399
	Pony club	0.936	4.066	<0.001	2.549	1.623 - 4.001
	Racing	-0.641	-1.683	0.092	0.527	0.250 - 1.111
	Sports	-0.907	-3.808	<0.001	0.379	0.230 - 0.624
Others	*n* other	0.004	1.04	0.298	1.004	0.996 - 1.013

Table S2- Results for ordinal regression assessing predictors of fear of fireworks in donkeys. Note: one level of each variable is a reference category.

**Donkeys**						
Variable	Level	Estimate	z-value	p-value	Odds ratio	95% CI
Sex	Female (entire)	Reference category				
	Male (entire)	-0.354	-1.050	0.294	0.702	0.362 - 1.360
	Male (castrated)	-0.400	-1.323	0.186	0.671	0.371 - 1.212
Age	≤5 years	Reference category				
	6 - 10 years	-0.259	-0.556	0.578	0.772	0.310 - 1.923
	11- 20 years	0.151	0.328	0.743	1.163	0.472 - 2.866
	>20 years	-0.365	-0.762	0.446	0.694	0.271 - 1.776
Origin	Breeder	Reference category				
	Family	0.814	1.797	0.072	2.258	0.929 - 5.490
	Other	1.124	2.302	0.021	3.079	1.182 - 8.020
	Rehomed	0.461	1.039	0.299	1.586	0.664 - 3.788
	Rescue	0.590	1.432	0.152	1.805	0.804 - 4.049
Yard	Farm	Reference category				
	Livery yard	-0.323	-0.730	0.465	0.724	0.304 - 1.723
	Other	-0.423	-0.970	0.332	0.655	0.279 - 1.539
	Private dwelling	-0.059	-0.153	0.878	0.943	0.443 - 2.004
Location	Rural	Reference category				
	Suburban	-0.016	-0.046	0.963	0.984	0.491 - 1.972
	Urban	0.060	0.150	0.881	1.062	0.486 - 2.317
Use						
	Other	-2.157	-1.652	0.098	0.116	0.009 - 1.494
	Pet/Companion	-0.681	-0.921	0.357	0.506	0.119 - 2.157
	Working	-0.929	-1.232	0.218	0.395	0.090 - 1.731
Size	Both	Reference category				
	Full	0.226	0.778	0.436	1.253	0.710 - 2.213
	Mini	0.262	0.670	0.503	1.299	0.604 - 2.793
Others	*n* others	-0.142	-2.428	0.015	0.867	0.773 - 0.973
